# A genome-scale metabolic model of a pathosystem sheds light on bacterial wilt

**DOI:** 10.1093/plphys/kiag428

**Published:** 2026-07-01

**Authors:** Léo Gerlin, Stéphane Genin, Caroline Baroukh

**Affiliations:** Univ Toulouse, INRAE, CNRS, LIPME, Auzeville CS 52627 31326 Castanet-Tolosan, France; INRAE, INSA Lyon, BF2I, UMR203, 11 avenue Jean Capelle Villeurbanne 69621, France; Univ Toulouse, INRAE, CNRS, LIPME, Auzeville CS 52627 31326 Castanet-Tolosan, France; Univ Toulouse, INRAE, CNRS, LIPME, Auzeville CS 52627 31326 Castanet-Tolosan, France

## Abstract

During plant infection, complex metabolic interactions occur between the host and the pathogen, including direct competition for resources. While pathogens exploit host-derived nutrients to sustain growth and virulence, plants attempt to restrict pathogen proliferation by limiting nutrient availability. To quantify the contribution of these trophic interactions to disease development, we developed a mathematical model of plant–pathogen metabolism. A genome-scale metabolic model of the pathogen was integrated with a genome-scale, multiorgan metabolic model of the plant and calibrated using experimental data. Model simulations were performed using a sequential flux balance analysis framework. This approach was applied to the *Ralstonia pseudosolanacearum*–tomato (*Solanum lycopersicum*) pathosystem. Quantitative fluxes of matter occurring during plant infection were predicted. The model shows that (i) plant photosynthetic capacity imposes a stronger constraint on bacterial proliferation than mineral availability; (ii) infection-induced reduction in plant transpiration first limits plant growth and subsequently restricts pathogen expansion; (iii) stem resource hijacking enhances bacterial growth but is likely limited; and (iv) pathogen-excreted putrescine is likely reutilized for the plant's needs. Together, these results provide a quantitative assessment of resource competition in plant–pathogen interactions and highlight the central role of water flow during infection by a fast-growing, xylem-colonizing bacterium.

## Teaser

An integrated plant–pathogen metabolic model shows that plant transpiration rate decline provokes plant growth arrest and limits bacterial proliferation during bacterial wilting.

## Introduction

Metabolism is a crucial parameter at the center of interactions between plants and pathogens. Indeed, during host infection, pathogens must efficiently find resources in hostile (and sometimes nutrient-poor) environments to sustain both their growth and virulence (Abu Kwaik and Bumann 2013). This implies a trade-off for the infecting pathogen, which must balance its carbon and energy expenditure between 2 objectives: on one hand, limiting plant defense and hijacking plant resources, and on the other hand, supporting its own multiplication and proliferation (Berger et al. 2007). On the plant side, a similar trade-off exists where the plant must distribute resources between maintenance/growth (to ensure reproduction) and immunity, while coping with an increasing infecting population that hijacks its metabolisms and affects its physiology (Herms and Mattson 1992; Walters and Heil 2007). Understanding the complex metabolic interactions occurring between the plant and the pathogen is essential for gaining a holistic view of the infection process. In particular, this knowledge could help to clarify (i) what drives the altered plant phenotype during infection (eg growth arrest), (ii) which environmental factors (light, minerals, etc.) limit pathogen growth and population density, (iii) whether the pathogens reprogram the host to allocate nutrients for its own benefit, as *Agrobacterium tumefaciens* does with opines (Zupan et al. 2000), and (iv) how plant metabolic pathways are affected by the presence of the pathogen, especially if the pathogen excretes metabolites. However, the use of a simple metabolomic comparison between infected and noninfected plants is not enough to answer these biological questions, in particular because pathogen metabolism is nearly indistinguishable from plant metabolism, except for very specific metabolites, and metabolism results from the dynamic interplay of multiple synthesis and degradation processes. To better tackle this interaction, a systemic and computational approach is necessary. Ideally, this approach should be quantitative, since only by assigning numerical values can one capture orders of magnitude, thresholds, and trade-offs that would otherwise remain invisible in purely qualitative reasoning.

To this end, we propose in this study to use genome-scale metabolic models (GSMMs). GSMMs integrate the metabolic potential of an organism of interest, encompassing thousands of metabolic reactions based on genomics, physiological, and biochemical evidences. These models generate quantitative estimations of how matter flows through the biological system under investigation (Gu et al. 2019) as well as phenotypic parameters such as growth. GSMMs have repeatedly proven to be pertinent tools for unraveling complex metabolism, making them suitable for elucidating plant–pathogen metabolic interactions (Gerlin et al. 2021c).

Recently, genome-scale metabolic modeling has been applied to the plant–pathogen system of tomato (*Solanum lycopersicum*) and *Phytophthora infestans* (Rodenburg et al. 2019). This approach enabled the identification of potential pathogen targets and plant pathways to engineer in order to block the infection process, but the model developed provided only qualitative insights, based solely on network connectivity and topology. Indeed, *P. infestans* biomass equation used equal stoichiometric coefficients, no mass conversion between plant and pathogen fluxes were used, unrestricted uptake of all metabolites from tomato cell cytosol by the pathogen was allowed, and calibration for maximal uptake rates for both *P. infestans* and the plant were missing. It also did not predict the effect of the presence of the pathogen on plant physiology.

A quantitative model of the plant–mutualist symbiont system *Medicago truncatula–Sinorhizobium meliloti* has also been recently developed (Pfau et al. 2018; diCenzo et al. 2020). Yet modeling a pathogenic interaction, as compared to a mutualistic one, raises additional methodological questions: (i) the choice of the objective function (ie the flux(es) to minimize/maximize) in an antagonistic relationship, given that the modeling approach assumes an optimal behavior of the biological system; (ii) the need for extensive data acquisition, including the plant/pathogen ratio, growth rates, and metabolites available for the pathogen. These considerations are crucial for developing accurate, meaningful, and quantitative models of plant–pathogen interactions.

In this study, we constructed the first quantitative genome-scale metabolic model of a plant–pathogen interaction. For our model pathosystem, we used *R. pseudosolanacearum* GMI1000 and its natural host, the tomato plant. *R. pseudosolanacearum* is a soil-borne β-proteobacterium that infects plants through the roots and subsequently colonizes the xylem vessels. Although xylem sap is generally considered a nutrient-poor environment (De La Fuente et al. 2022), the pathogen proliferates extensively within this niche and rapidly spreads from vessel to vessel, leading to systemic colonization of the aerial parts of the plant. Intense colonization of xylem vessels has been reported, along with, to a lesser extent, radial expansion into the apoplast of pith and cortical tissues (Planas-Marquès et al. 2020). This massive proliferation results in very high bacterial densities within the stem, ultimately causing partial or complete occlusion of xylem vessels (Ingel et al. 2022). Consistent with these observations, plants exhibit a progressive reduction in growth and transpiration (Gerlin et al. 2021a), with symptoms emerging at bacterial densities ranging from 10^6^ to 10^8^ colony-forming units per gram of fresh stem weight (CFU/g stem FW). During infection, *R. pseudosolanacearum* displays a rapid growth phase up to approximately 10^8^ CFU/g stem FW, followed by a marked slowdown until reaching 10^9^ to 10^10^ CFU/g stem FW. Several studies report a maximal bacterial load of around 10^10^ CFU/g stem FW (see, for example, [Grimault et al. 1994; Lowe et al. 2015]). Although the precise cause of plant death remains unclear, vascular occlusion resulting from high bacterial densities in the xylem is considered a primary driver of lethal wilting symptoms. In addition, competition for limited nutrients within the xylem sap between the host and the pathogen may further contribute to disease progression.

R. *pseudosolanacearum* has been extensively studied for its virulence factors (Vailleau and Genin 2023) and has gained growing attention for the metabolic aspects of its lifestyle (Peyraud et al. 2016;Lowe-Power et al. 2018b; Peyraud et al. 2018; Gerlin et al. 2021a; Hamilton et al. 2021). It was among the first plant pathogens having a high-quality GSMM (Peyraud et al. 2016). GSMMs for the tomato plant, *R. pseudosolanacearum*'s natural host, were also developed: a cell genome-scale metabolic model was originally published in 2016 (Yuan et al. 2016), and a genome-scale multiorgan metabolic model, with particular emphasis on xylem vessels (where *R. pseudosolanacearum* is most metabolically active), was recently published (Gerlin et al. 2022). A core metabolic model of tomato stem in the context of a fungal infection has also been developed (Lacrampe et al. 2024).

The quantitative genome-scale metabolic model developed in this study was named VIRION for VIrtual *Ralstonia*-tomato plant interactION. It was based on the multiorgan metabolic model of the tomato plant, representing different plant organs during vegetative growth (leaf, stem, root, with xylem and phloem as exchange compartments), combined with *R. pseudosolanacearum* model represented as an additional compartment connected to the xylem. To accurately simulate the antagonistic interaction between the plant and the pathogen, we employed a set of sequential flux balance analyses (FBAs). To be more accurate, VIRION also integrates a comprehensive set of physiological and metabolic data: (i) tomato vegetative growth (organ ratio, growth rates, xylem metabolites), (ii) tomato infection by *R. pseudosolanacearum* (plant/pathogen ratios, impacts of infection on physiology), and (iii) *R. pseudosolanacearum* physiology and metabolic capacities (growth rates, assimilation and excretion capacities on xylem metabolites).

The model only focused on the trophic aspect of metabolism. It did not account for the plant's immune response or the pathogen's virulence, as modeling such phenomena is highly challenging and requires alternative approaches. The model allows quantitative estimation of *R. pseudosolanacearum* growth capacity in planta at different bacterial loads, as well as the pathogen’s impact on plant growth and metabolism. We used the model to analyze factors causing the infected plant to cease growth and those limiting bacterial growth. Finally, we also investigated potential hijacking of stem resources during the disease, and the fate of the bacterial excreted putrescine in the plant.

## Results

### Modeling an antagonist plant–pathogen interaction using a multicompartment metabolic model and sequential FBAs

**Figure 1:**
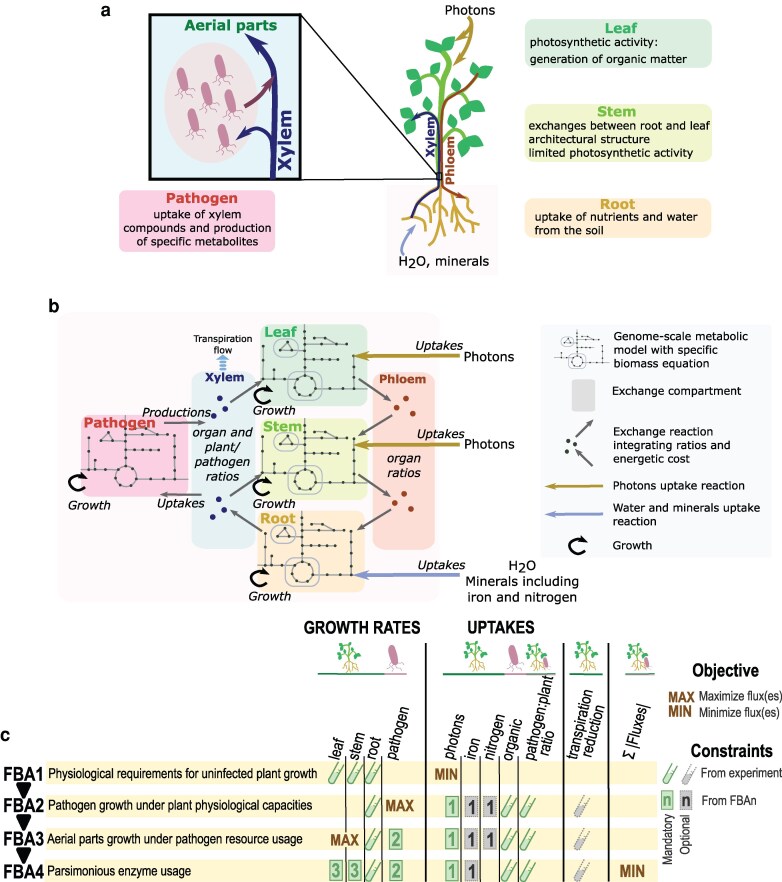
Schematic representation of the metabolic model VIRION. a) The main components of the system modeled in this study. Leaf, stem, root, and the pathogen are each represented by a genome-scale metabolic model. Xylem and phloem are exchange compartments. Inputs of the system are photons, connected to leaf and stem, and water and minerals, connected through the root. b) Schematic representation of the multicompartment model and quantitative approach used in this study. Arrows represent relationships between the different organs and the possible quantitative constraints that can be set in the model (growth, uptakes, exchanges, transpiration flow). c) Sequential flux balance analyses (FBAs). Schematic representation of the different FBAs solved in a sequential approach to obtain a phenotype prediction of the pathosystem (growth rate of plant organs and pathogen) as well as fluxes of matter. The constraints from experiments are detailed in Table S1.

To model the metabolic interaction between *R. pseudosolanacearum* and the tomato plant, we represented the system as 4 metabolic compartments corresponding to the main metabolic roles of the plant (leaf, stem, root) and the pathogen. Metabolites can be exchanged between these compartments through xylem and phloem (Fig. 1a). Each of these 4 compartments was represented as a GSMM: iRP1476 for the pathogen (Peyraud et al. 2016) and Sl2183 for the plant compartments (Gerlin et al. 2022) (Fig. 1b), with minor modifications as reported in the Methods section. As in Gerlin et al. (2022), the physiological roles of each compartment (described in Fig. 1a) were implemented as FBA constraints (Fig. 1b). In brief: (i) minerals and water can enter the system only through the root and are transported to the upper parts of the plant and to the pathogen exclusively via xylem, (ii) photons are assimilated in both the leaf and the stem, with higher efficiency in the leaf, (iii) the carboxylase/oxygenase activity of the Rubisco was constrained to experimentally measured values. For a complete description and justification of these constraints, readers are referred to Gerlin et al. (2022).

FBA is an approach based on an optimization problem (typically formulated as linear programming), in which one or several objective reactions are maximized or minimized to compute metabolic fluxes (Orth et al. 2010). Usually, when modeling ecosystems, the organisms composing the system are assumed to collectively maximize global biomass (diCenzo et al. 2020). However, this assumption is not appropriate for our antagonistic system, in which the pathogen grows at the expense of the plant, leading to decoupled growth dynamics between the 2 partners. Moreover, in our system, the metabolic composition of xylem sap is severely altered upon bacterial infection, particularly at high bacterial densities (Gerlin et al. 2021a), thereby challenging the quasi-steady-state approximation (QSSA). To model this antagonistic interaction, we approximated the system using a sequence of 4 FBAs (Fig. 1c; Table S1), each solved at a given bacterial density under the assumption of a QSSA. In this framework, the initial FBA identifies one or more optimal fluxes, which are then propagated as additional constraints into the subsequent FBA. This procedure is iterated 3 times, resulting in a 4-step FBA workflow. The workflow is repeated across a range of bacterial densities comprising those observed *in planta* (Gerlin et al. 2021a), enabling the prediction of both bacterial and host metabolic states during infection. Bacterial density is incorporated by scaling the stoichiometric coefficients of exchange reactions between the xylem and the pathogen at each density (see Methods for details).

The sequential 4-FBA approach consisted of the 4 following steps:

FBA 1: Estimation of the requirements of a healthy plant in terms of photons, nitrogen, and other minerals necessary for an optimal growth of the leaf, stem, and root.

FBA 2: Estimation of pathogen growth while constraining plant photon and mineral inputs to the levels obtained in FBA 1. This ensured that resource consumption did not exceed that of a healthy plant. Photon constraints were always applied, whereas mineral constraints were optional. In addition, the transpiration flow—known to be significantly reduced after pathogen inoculation (Gerlin et al. 2021a) and influence xylem exchanges—could be constrained at this step (see Methods for details).

FBA 3: Estimation of leaf and stem growth rates given the pathogen growth determined in FBA 2. In both FBA 2 and FBA 3, the root growth rate was fixed to experimentally observed values, since root growth is minimally affected by xylem colonization according to experimental data on infected tomato plants (Gerlin et al. 2021a).

FBA 4: Estimation of metabolic fluxes in the pathosystem under a parsimonious enzyme usage constraint. This final simulation yielded the flux distribution for a given bacterial density.

From FBA 2 onward, unless otherwise specified, fluxes from the stem to the xylem were forbidden, since these fluxes were null in FBA1 (input requirements for a healthy plant). These 4 FBAs were performed for bacterial densities ranging from 10^6^ to 10^12^ CFU/g stem FW, which are bacterial densities typically measured in the plant during an infection cycle (Gerlin et al. 2021a). The outputs of the 4 FBAs consist of metabolic fluxes within the pathosystem (ie the pathogen, plant organs, and xylem and phloem saps), as well as the growth rate of both the pathogen and the plant for each bacterial density simulated. These simulations’ outputs were compared with experimental observations to validate the model and gain insight into the pathosystem (Gerlin et al. 2021a). In particular, we assessed whether the model correctly captures (i) the bacterial density at which plant growth declines to zero (starting around 10^8^ CFU/g stem FW), (ii) the bacterial density at which the pathogen transitions from high growth rate (until 10^8^ CFU/g stem FW) to an intermediate growth rate (from 10^8^ CFU/g stem FW to 10^10^ CFU/g stem FW) to a null growth rate (above 10^10^ CFU/g stem FW) (Figure S2c).

### Identifying metabolic bottlenecks during the plant–pathogen interaction

**Figure 2:**
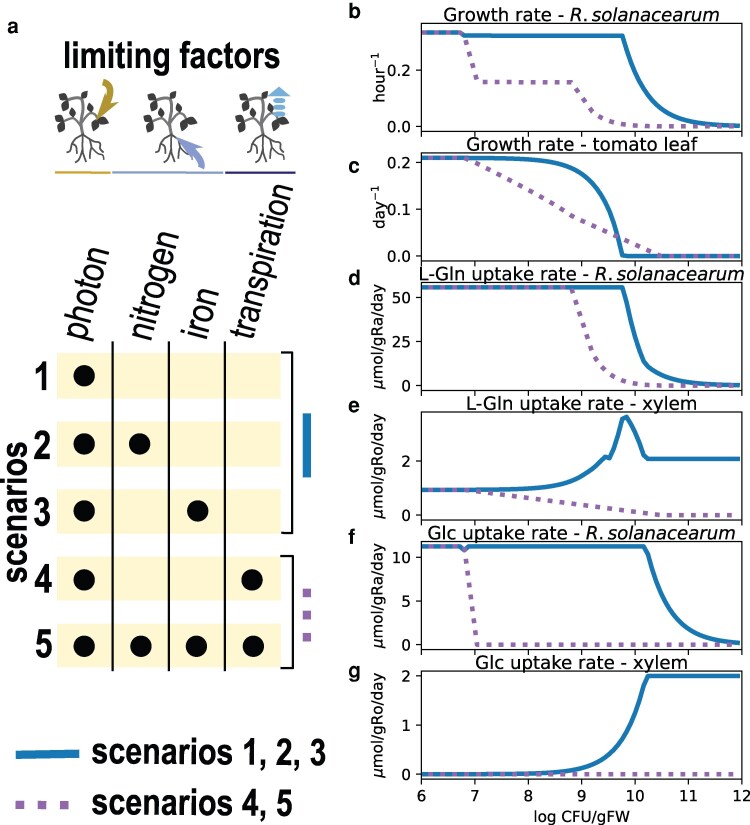
Effect of plant environmental conditions on growth and metabolic exchanges in the pathosystem. a) Simulation scenarios in which different plant environmental conditions were constrained at the FBA 2 step during sequential FBAs. The upper bounds of photons, nitrogen, and iron were set to the values obtained from FBA 1. Transpiration constraint was implemented as constraints on the upper bound of fluxes from root to xylem using a linear relationship computed using the experimental values of Gerlin et al. (2021a). b to g) Effects of plant environmental conditions on growth and metabolic exchanges for bacterial densities ranging from 10^6^ to 10^12^ CFU/g stem FW. b) *R. pseudosolanacearum* growth. c) Tomato leaf growth. d) Glutamine uptake rate by *R. pseudosolanacearum*. e) Glutamine transport rate from root to xylem sap. f) Glucose uptake rate by *R. pseudosolanacearum*. g) Glucose transport rate from root to xylem sap. gRa: dry weight grams of *R. pseudosolanacearum*, gRo: dry weight grams of root, L-Gln: L-glutamine, Glc: D-glucose, CFU: colony-forming units, FW: fresh weight.

We used the VIRION model to assess which plant environmental factors—light, nitrogen, iron, or water—constrained the plant–pathogen interaction. The analysis considered these factors individually and in combination (Fig. 2a).

From FBA 2 onward, maximal light uptake is constrained to the value found from FBA 1 (minimization of plant photons for a healthy tomato plant, and growth rate constrained to experimental values of a healthy plant). Because infected plants displayed reduced leaf area compared to healthy ones (Gerlin et al. 2021a), light constraint was treated as mandatory: an infected plant cannot absorb more photons than a healthy plant.

When light is the sole limiting factor (Fig. 2a, scenario #1), thereby constraining photosynthesis and organic carbon production, *R. pseudosolanacearum* grows at its maximal rate up to a density of 10^9.7^ CFU/g stem FW (Fig. 2b). Beyond this threshold, bacterial growth declines sharply, falling below 0.01 h^−1^ once densities exceed 10^11.2^ CFU/g stem FW. These results indicate that if *R. pseudosolanacearum* relies exclusively on biotrophic growth sustained by xylem sap metabolites, the plant cannot support bacterial proliferation beyond roughly 10^11^ CFU/g stem FW. Analysis of the simulated substrate uptake fluxes reveals that glutamine is the primary carbon source sustaining *R. pseudosolanacearum* growth (Fig. 2d). Glutamine uptake remains high up to approximately 10^10^ CFU/g stem FW, beyond which it becomes limiting—first for the growth of the plant's aerial tissues, and subsequently for *R. pseudosolanacearum*. This limitation is temporarily alleviated by glucose uptake, a secondary carbon source that is co-consumed with glutamine. However, at slightly higher bacterial densities (10^10.25^ CFU/g stem FW), the maximal glucose uptake flux in the xylem is also reached, further constraining bacterial growth.

Part of these simulation results are consistent with experimental observations: *in planta* analyses and in vitro assays have identified glutamine as the principal carbon source of *R. pseudosolanacearum* in tomato xylem sap (Gerlin et al. 2021a; Baroukh et al. 2022, 2025). While glucose can moderately support bacterial proliferation, it has been shown to play only an accessory role (Gerlin et al. 2021a; Baroukh et al. 2022, 2025). However, plant growth capacities were overestimated by the simulations results: optimal leaf and stem growth were maintained in silico up to a bacterial density of 10^8.5^ CFU/g stem FW (Fig. 2c), whereas growth arrest of plant aerial parts was experimentally observed between 10^6^ and 10^8^ CFU/g stem FW (Gerlin et al. 2021a). In addition, experimental data also show that the bacterial growth rate is intermediate from 10^8^ CFU/g stem FW, which is not the case in our model, with a maximal growth rate until 10^9.7^ CFU/g stem FW (Gerlin et al. 2021a) (Figure S2a and c). Finally, maximal bacterial density observed in many experiments involving *R. pseudosolanacearum* have shown a maximal density just below 10^10^ CFU/g stem FW (see, for example, [Grimault et al. 1994; Lowe et al. 2015]), but the model predicted 10^11^ CFU/g stem FW. These discrepancies indicate that additional factors other than light contribute to plant growth arrest and pathogen decreased growth rate. We thus investigated the role of minerals’ limitation on the pathosystem behavior.

From FBA 2 onward, maximal nitrogen and iron plant uptake rate was constrained on the rates found from FBA 1 (Fig. 2a, scenarios #2 and #3). While other minerals could be studied, our study focused on nitrogen and iron. Nitrogen source affects the concentration of xylem organic metabolites in tomato (Bialczyk et al. 2004), and both nitrogen and iron were suggested to influence the switch to virulence in *R. pseudosolanacearum* (Bhatt and Denny 2004; Dalsing and Allen 2014; Dalsing et al. 2015). When mineral constraints—specifically iron and nitrogen—were applied, either separately or together, the simulation outcomes were similar to those obtained under light limitation alone (Fig. 2). Nitrogen and iron therefore are less stringent bottlenecks than light. Yet the model still exhibited the same discrepancies when compared to experimental data, indicating that additional factors other than light and minerals contribute to plant growth arrest and pathogen decreased growth rate. We thus investigated the role of transpiration on the pathosystem behavior.

Transpiration was constrained by limiting the upper bound of root to xylem transport fluxes (Fig. 2a, scenario #4), based on a previously established linear relationship between *in planta* bacterial density and transpiration reduction (Gerlin et al. 2021a) (see Methods for further details). Limiting transpiration flow led to a more stringent bottleneck than light and minerals, with a decline in both plant and *R. pseudosolanacearum* growth rates occurring at lower bacterial densities (Fig. 2b and c). Indeed, from approximately 10^7^ CFU/g stem FW, reduced transpiration limited the availability of xylem sap nutrients for both the bacterium and the aerial tissues. Because the model prioritizes *R. pseudosolanacearum* growth at the expense of that of the plant, an immediate effect on plant development was observed (Fig. 2c). At higher bacterial densities, xylem fluxes became also insufficient to sustain *R. pseudosolanacearum* rapid growth, resulting in an intermediate growth rate (Fig. 2b) from 10^7^ to 10^9^ CFU/g stem FW before a drastic decline to a near-null growth rate above 10^10^ CFU/g stem FW. These results are consistent with what was observed experimentally in Gerlin et al. (2021a), with a maximal growth rate until 10^8^ CFU/g stem FW, and an intermediate growth rate between 10^8^ to 10^9.5^ CFU/g stem FW and a maximal bacterial density just below 10^10^ CFU/g stem FW (Figure S2a and c). Looking at xylem fluxes, glutamine remained abundant enough to satisfy the maximal uptake rate of *R. pseudosolanacearum* (Fig. 2d) until 10^9^ CFU/g stem FW while other metabolites present at much lower concentrations in the xylem—such as glucose (Fig. 2f), sucrose, and amino acids (Figure S1)—were no longer assimilated or only at reduced rates from 10^7^ CFU/g stem FW, explaining the intermediate growth phase observed. Above 10^9^ CFU/g stem FW, glutamine became limiting, severely impairing bacterial growth and rapidly leading to growth arrest.

Finally, adding nitrogen and iron limitations to the model—already constrained by photon availability and transpiration—did not markedly alter the simulated progression of the disease (scenario #5, Fig. 2). This result suggests that decline in plant transpiration, which reduces xylem fluxes, exerts the strongest influence on both plant and pathogen metabolic capacities. Transpiration decline is therefore the most probable mechanism underlying plant growth arrest, pathogen growth arrest, and the establishment of maximal bacterial density. In contrast, limitations in nitrogen, iron, or carbon (via photon limitation) do not seem to represent the primary drivers of these experimental observations.

### Assessing if hijacking tomato stem resources is a major pathogen strategy

In the previous simulations, the contribution of stem metabolism to xylem sap was not permitted since the fluxes were all null in the simulation results of FBA 1 (estimation of the requirements of a healthy plant in terms of photons, nitrogen, and other minerals necessary for an optimal growth of the leaf, stem, and root). However, such fluxes might occur in an infected tomato plant if the bacterium employs a host resource hijacking strategy modulating actively metabolic fluxes in cells adjacent to the loci of the infection. This idea aligns with the proposed model that certain type III secretion effectors of *R. pseudosolanacearum* can modify plant metabolism (Macho 2016; Landry et al. 2020).

**Figure 3:**
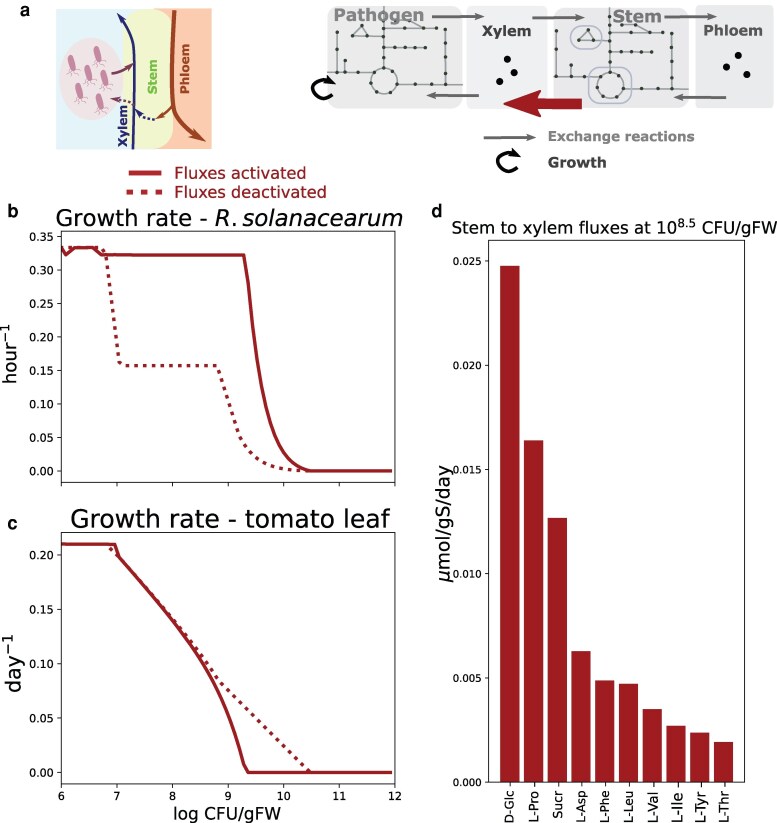
Effect of xylem sap enrichment by the stem on plant growth, pathogen growth, and xylem sap composition. a) Schematic representation of the investigated plant fluxes. In scenarios #1 to #5, the stem could not enrich xylem sap. This constraint was lifted to assess its effect on the metabolism of the pathosystem. b) Pathogen growth rate when the stem can (line) or cannot (dotted line) enrich xylem sap for bacterial densities ranging from 10^6^ to 10^12^ CFU/g stem FW. c) Plant growth rate when the stem can (line) or cannot (dotted line) enrich xylem sap for bacterial densities ranging from 10^6^ to 10^12^ CFU/g stem FW. d) Fluxes of matter from stem to xylem when the stem can enrich xylem sap. Fluxes were computed for a bacterial density of 10^8.5^ CFU/g stem FW. Fluxes are 0 when stem cannot enrich xylem sap. gS: grams of stem dry weight, D-Glc: D-glucose, L-Pro: L-proline, Sucr: sucrose, L-Asp: L-aspartate, L-Phe: L-phenylalanine, L-Leu: L-leucine, L-Val: L-valine, L-Ile: L-isoleucine, L-Tyr: L-tyrosine, L-Thr: L-threonine, FW: fresh weight.

We thus re-modeled scenario #5 from the previous section (where photons, nitrogen, iron, transpiration limitations were applied; Fig. 2a), this time allowing fluxes from the stem to the xylem, creating scenario #6 (Fig. 3a). Simulation results indicated that letting the stem enrich xylem sap had only a slight effect on tomato leaf growth (Fig. 3c). In contrast, a more pronounced effect was observed on pathogen growth (Fig. 3b): *R. pseudosolanacearum* could maintain a maximal growth rate up to 10^9.2^ CFU/g stem FW, compared to 10^7^ CFU/g stem FW in scenario #5 (Fig. 3b). This enhanced capacity for growth at higher bacterial densities was due to the enrichment of xylem sap in glucose, sucrose, and amino acids, facilitated by the stem's contribution (Fig. 3d; Figure S1). In these simulation results, nutrients from the stem thus partially counterbalanced the effect of reduced transpiration and allowed *R. pseudosolanacearum* to reach higher densities. However, the plausibility of such extensive rewiring of stem fluxes by the pathogen is questionable in light of experimental data. Indeed, bacterial population dynamics measured *in planta* showed an intermediate growth rate of *R. pseudosolanacearum* above 10^8^ CFU/g stem FW (Gerlin et al. 2021a), aligning more closely with scenario #4 or #5 (without stem resource hijacking) than with scenario #6 (Figure S2b and c).

### The fate of putrescine in the *Ralstonia*–tomato interaction

**Figure 4:**
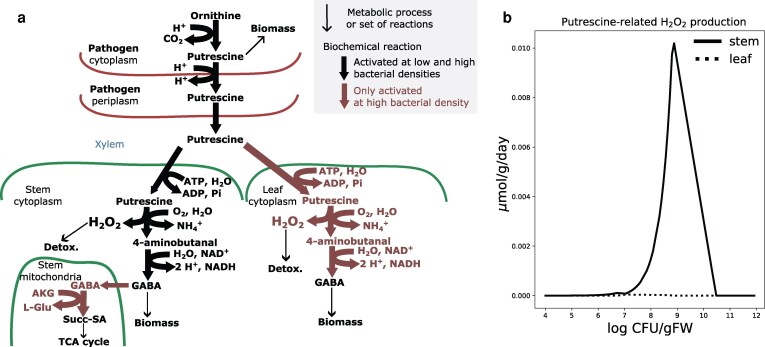
Fluxes predictions surrounding putrescine in VIRION. a) Metabolic pathways involving putrescine synthesis and degradation in both the pathogen and the plant. Only fluxes activated between putrescine production by the pathogen and its assimilation by the plant are represented. b) Prediction of putrescine-related H_2_O_2_ production in stem (solid line) and leaf (dotted line) for bacterial densities ranging from 10^6^ to 10^12^ CFU/g stem FW. On the *y* axis, “g” means either “g of stem dry weight (solid line)” or “g of leaf dry weight (dotted line).” FW: fresh weight.

Putrescine fate during plant–*R. pseudosolanacearum* interaction is frequently questioned, as putrescine is abundantly produced by the bacterium in vitro and *in planta* (Peyraud et al. 2016; Lowe-Power et al. 2018a; Gerlin et al. 2021a) and contributes to virulence by accelerating wilting symptoms (Lowe-Power et al. 2018a). We analyzed predicted fluxes of putrescine in our model including its production by *R. pseudosolanacearum*—calibrated to its experimentally measured excretion rate in a xylem-mimicking medium (Baroukh et al. 2025)—and its assimilation by the plant at low and high bacterial densities (10^6.5^ and 10^8.5^ CFU/g stem FW) (Fig. 4a).

When putrescine is produced by the bacterium from ornithine, a small portion is incorporated into the bacterial biomass, while the majority is exported to xylem (Peyraud et al. 2016; Baroukh et al. 2022, 2025). Subsequently, the plant aerial tissues can assimilate part of this excreted putrescine. Our model predicted that at low bacterial density, all putrescine is converted into 4-aminobutanal and then into gamma-aminobutyric acid (GABA). The amount of GABA produced from putrescine at this bacterial density is insufficient to meet the stem biomass requirements for GABA, which are supplemented by other reactions producing GABA from glutamate (glutamate decarboxylase, R_GUDC, EC 4.1.1.15). At high bacterial density, GABA production from putrescine exceeds the stem biomass requirements. This excess is accommodated by: (i) activation of the GABA shunt pathway allowing incorporation of GABA into TCA cycle via transport into mitochondria (R_4abut_tx_m_) and conversion into succinate-semialdehyde (4-aminobutyrate transaminase, R_ABTArm) in the stem, (ii) activation of the pathway enabling conversion of putrescine into GABA and its assimilation into the leaf. Other reactions associated with putrescine degradation such as conversion into N-acetylspermidine (N1-acetylspermidine oxidase, identifier R_RE1537C in the model) or into N-methylputrescine (putrescine N-methyltransferase, R_PMT) were not predicted to carry fluxes, because these reactions produce dead-end metabolites not connected to central metabolism. Interestingly, excess putrescine assimilated by the plant via GABA leads to mandatory production of hydrogen peroxide (H_2_O_2_) (Fig. 4b), a key modulator of redox homeostasis (Gerlin et al. 2021b), particularly in the stem. H_2_O_2_ production follows pathogen growth, peaking at approximately at 10^9^ CFU/g stem FW. At higher bacterial densities, the pathogen's limited growth capacity reduces putrescine production flux, thereby decreasing H_2_O_2_ flux.

## Discussion

### Metabolic modeling of a plant–pathogen interaction: keys to success

Mathematical modeling can help to understand the complex metabolic interactions occurring between the plant and the pathogen, which is essential for gaining a holistic view of the infection process. In the present study, we developed VIRION, a GSMM predicting in a quantitative manner the metabolic fluxes of the plant, the pathogen, and their metabolic exchanges along with the growth rate of each organism. VIRION was built by (i) merging the metabolic networks of *R. pseudosolanacearum* and tomato plant, (ii) using a sequential FBA simulation framework, and (iii) using a comprehensive set of physiological and metabolic data from Gerlin et al. (2021a) to calibrate and validate the model.

VIRION was able to illustrate its relevance by predicting correctly the growth rates of the aerial part of the plant and the pathogen close to what was experimentally observed including the intermediate growth rate of the bacteria at intermediate to high bacterial densities. VIRION also allowed the dissection of previously unquantified aspects of the *R. pseudosolanacearum*–tomato interaction such as the following: (i) the plant's photosynthetic capacity imposes a stronger constraint on bacterial proliferation than mineral availability, (ii) a reduction in plant transpiration limits and ultimately stops plant and pathogen growths, (iii) hijacking stem resources, potentially through virulence factors, can enhance bacterial growth but is not essential for *R. pseudosolanacearum*, and (iv) pathogen-excreted putrescine is predicted to be directly reused for plant biomass.

In VIRION, we chose to use FBA because it is a methodology widely used in the systems biology community, thanks to its simplicity to set up, its capacity to model at the genome-scale level, and its relatively low number of parameters to calibrate (Orth et al. 2010). However, this methodology relies on the quasi-steady-state assumption (QSSA), assuming that no internal metabolites of the system accumulate or get depleted, as well as the fact that the system is optimal with respect to an objective (most often assumed to be growth). The difficulty in modeling a plant–pathogen system lies in the fact that the pathogen grows at the expense of the plant, implying antagonistic objectives between the organisms composing the system. Therefore, questions arise regarding the choice of the objective function that should be optimized. In addition, the pathogen might deplete or accumulate some metabolites upon its growth in the plant, which probably challenges the QSSA.

As a first approximation to quantitatively model a plant–pathogen interaction, we proposed the use of sequential FBAs at several bacterial densities. We thus assumed that for each bacterial density, the internal metabolites were “at steady-state.” We also assumed that pathogen growth was at the expense of the aerial parts of the plant. Despite these strong hypotheses, VIRION was able to correctly predict what was experimentally observed. However, the model could be enhanced from a methodological point of view by slightly relaxing the QSSA on the metabolites composing the xylem sap. This would allow modeling dynamically the infection process and predicting in a more biologically relevant manner the plant–pathogen interaction.

The success of VIRION also relied on a comprehensive set of experimental data used for model calibration and validation. First, organ mass ratios and growth rates in healthy tomato plants were essential to ensure that resource allocation accurately reflects the physiological contributions and metabolic demands of each organ at the whole-plant level. This enabled a realistic representation of source–sink relationships among organs and the resulting xylem sap composition. Secondly, accounting for mass ratios between the plant and the pathogen is likewise critical to maintain biological realism. The relative biomass of each partner directly constrains the magnitude of metabolic fluxes they can sustain; neglecting these differences can lead to an overestimation of one partner's contribution relative to the other. Incorporating these ratios ensures that nutrient exchange, growth rates, and metabolic demands are properly scaled to the actual proportions of host and pathogen. Thirdly, using experimentally determined growth rates of *R. pseudosolanacearum* in a xylem-mimicking medium was essential to constrain pathogen proliferation within biologically realistic bounds in the host environment. Integrating its measured assimilation and excretion capacities allowed the model to accurately capture nutrient uptake and putrescine release, key processes underlying the infection process and improving the reliability of model predictions. Finally, it was important to account for the infection-induced decrease in plant transpiration, as this process is a major driver of plant growth arrest and the attainment of maximal bacterial densities during infection.

One limitation, however, is that the dataset was derived from a single study; while it reflects a typical infection by *R. pseudosolanacearum*, it may not capture the full diversity of metabolic interactions that occur in other host plants cultivars or environmental contexts. As a direct perspective, the model could be used to study the impact of plant environmental conditions on the plant–pathogen interaction.

### Modeling the interplay between bacterial virulence and plant immunity

VIRION only focused on the trophic aspect of metabolism. It did not account for the plant's immune response or the pathogen's virulence, as modeling such phenomena is highly challenging and requires alternative approaches such as dynamic gene regulatory networks or functional association networks (eg immunity-related metabolomic measurements) that are not available for our pathosystem. Moreover, the present study addressed an infection stage that, in the case of *R. pseudosolanacearum*, corresponds to intense multiplication within plant tissues, and thus likely occurs once most of the immune defenses have already been overcome (Gerlin et al. 2021a; Baroukh et al. 2025). At this stage, trophic interactions are expected to be predominant. In addition, this stage of the interaction has received little attention so far, whereas numerous studies have focused on early plant immunity, such as perception and signaling (DeFalco and Zipfel 2021; Jian et al. 2024). Even if VIRION did not model explicitly immunity and virulence, it could still successfully evaluate some virulence-related aspects such as the impact of plant resource diversion by *R. pseudosolanacearum* or the impact of its putrescine excretion on the plant H_2_O_2_ production. VIRION can thus be seen as a starting point for the development of more complex plant–bacteria interaction models that explicitly integrate the interplay between metabolism, immunity, and virulence mechanisms.

Incorporating explicitly the impacts of virulence and immunity on flux distribution could avoid a potential underestimation of nutrient requirements by the plant or the pathogen. As a first approach, one could calibrate a virulence cost on the pathogen side as well as an immunity cost on the plant side, both as equivalent mol of ATP. These terms would allow in rough manner to integrate the cost of immunity and virulence in VIRION. However, additional data to calibrate these terms would be necessary, such as the gain of growth rate for an avirulent *R. pseudosolanacearum* mutant and a tomato mutant defective in immunity. One could also model in a more mechanistic manner immunity and virulence by better representing all the immune- and virulence-related compounds in the metabolic networks of the tomato and of *R. pseudosolanacearum*. *R. pseudosolanacearum* metabolic network already comprises a virulence network composed of many virulence-related metabolites such as type II and type III effectors or exopolysaccharides and would only need to be updated to latest discoveries related to its virulence (Peyraud et al. 2016). Concerning the plant side, the tomato metabolic network could be updated, thanks to literature as well as the recently published potato genome-scale metabolic model comprising immunity-related metabolites (Zrimec et al. 2025). However, extensive metabolomics data for both tomato immune metabolites and *R. pseudosolanacearum* virulence metabolites will be necessary to perform quantitative predictions, as well as extensive characterization of the related macromolecules (eg effectors, biofilm elements, antimicrobial peptides). Some qualification and quantifications of exopolysaccharides and their impact on growth were already done (Peyraud et al. 2016), but a full quantification of virulence and immunity-related metabolites remains a challenging perspective. Finally, on a broader perspective, it would be relevant to add the regulatory layer to such model, to better represent triggering of immunity on the plant side and virulence on the pathogen side, as well as dose–effect responses that are difficult to represent in metabolic modeling. This is much more challenging since it requires the hybridization of discrete-regulatory-type models with continuous metabolic models (Liu and Bockmayr 2020; Thuillier et al. 2022).

### Using mathematical modeling to better understand bacterial wilt disease

Since 2016, several mathematical models have been developed to better understand the biology behind bacterial wilt disease. The first model, released in 2016, consisted of a high-quality metabolic network of *R. pseudosolanacearum* simulated in diverse in vitro environments using FBA (Peyraud et al. 2016). This model allowed measuring the ATP-equivalent cost of EPS synthesis and virulence. This metabolic network was subsequently interfaced with a regulatory network controlling multiple virulence factors and simulated across a broad set of environmental conditions using a discrete logical modeling method (Peyraud et al. 2018). The resulting model revealed that the virulence regulatory network exerts a control of the primary metabolism to promote robustness upon infection.

More recently, Baroukh et al. (2025) modeled the *in planta* population dynamics of *R. pseudosolanacearum* during tomato infection using a macroscopic modeling framework relying on Monod kinetics. This mathematical model predicted substrate consumption, putrescine production, and biomass growth of *R. pseudosolanacearum*. Unlike previous models, it did not incorporate the metabolic network of *R. pseudosolanacearum* or its regulatory network, nor did it include the plant's metabolic network, which was simply modeled as a continuous environment providing substrate fluxes to the pathogen. Despite these simplifications, the model accurately reproduced experimental observations, including high bacterial densities and the depletion of glutamine and asparagine. Additionally, it estimated key parameters such as the bacterial mortality rate within the plant and the rate at which bacterial putrescine is assimilated by the plant.

In the present study, the metabolic networks of *R. pseudosolanacearum* and the tomato plant were merged, and the resulting model was simulated using sequential FBAs for diverse bacterial densities within the plant. The model demonstrated several key biological insights such as transpiration as the main factor responsible for plant growth arrest and *R. pseudosolanacearum* maximal density in the plant.

In each of the *R. pseudosolanacearum* mathematical models developed so far, different modeling approaches have been used, different objects have been modeled, various experimental data have been used for calibration, and distinct biological questions have been addressed. Consequently, each model possesses its own specificity and provides unique insights into the mechanisms at play during bacterial wilt disease. Future directions could involve improving existing models by better representing the plant putrescine degradation pathway. Alternatively, models could be made more complex by considering the spatial aspects of infection and representing the effects of abiotic and biotic conditions of the plant on the pathosystem dynamics.

### Plant transpiration is the main factor leading to growth arrest of both the host and the pathogen wilt disease

**Figure 5:**
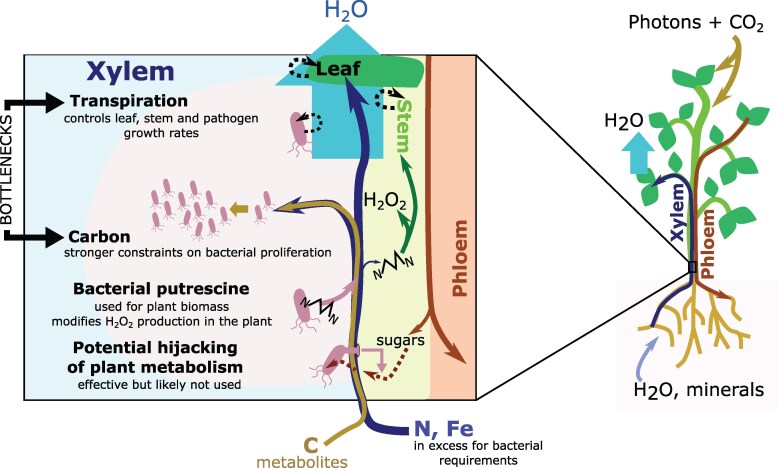
Key metabolic parameters during bacterial wilt at the origin of a successful infection. Decrease in transpiration during infection appears to be the most likely mechanism behind the cessation of plant growth and pathogen growth. Among diverse nutrients, carbon imposes a stronger constraint on bacterial proliferation than mineral availability. The study also showed that, although diverting nutrients from the phloem to the xylem via the stem is an effective strategy for increasing bacterial multiplication in plants, it is likely used in a limited manner in the case of bacterial wilt. Finally, the effect of bacterial putrescine production on host metabolism was shown to interfere with H_2_O_2_ synthesis in the plant, likely having an effect on reactive oxygen species (ROS) response.

VIRION allowed testing and identifying which physiological or environmental factors are behind plant and pathogen growth arrests during bacterial wilt disease. First, our simulation results showed that the maximal carbon uptake capacity of the plant (through photosynthesis) imposes a stronger constraint on *R. pseudosolanacearum* proliferation than mineral availability cells (Fig. 5). However, we also found that carbon, or any other metabolic bottlenecks such as nitrogen and iron, are insufficient to explain the early arrest of plant growth that was experimentally observed in Gerlin et al. (2021a). To explain plant growth arrest, limiting transpiration was necessary. This restriction reduces xylem sap movement, limiting nutrient delivery first to the aerial plant tissues and subsequently to the pathogen (Fig. 5). Interestingly, at densities where this transpiration-limitation is induced, the plant growth was affected immediately, in agreement with experimental observations, contrary to the bacteria which continued to thrive, even if at an intermediate rate, up to several logs higher. This underlines the pathogen's perfect metabolic adaptation to this challenging environment.

Naturally, questions arise on the mechanism responsible for plant transpiration decrease upon *R. pseudosolanacearum* presence in xylem vessels. Physical presence of *R. pseudosolanacearum* cells could impede xylem flow in itself, but an indirect mechanism such as large bacterial exopolysaccharides excretion could also be responsible by making xylem sap so viscous that it impedes its flow (Ingel et al. 2022; Cope-Arguello et al. 2026). To discriminate between these hypotheses or others possible mechanisms, further investigation is necessary.

### Stem resource hijacking, a strategy likely used minimally by *R. pseudosolanacearum* during tomato infection

In the literature, xylem sap is commonly described as a nutrient-poor environment that cannot sustain the biotrophic growth of a plant pathogen to high densities (De La Fuente et al. 2022), thereby requiring the bacterium to force the plant to release additional nutrients, such as sugars (MacIntyre et al. 2020; Hamilton et al. 2021). The mechanisms and the extent to which such mechanisms could contribute to enhance *R. pseudosolanacearum* growth *in planta* were not elucidated yet. In particular, it could be a passive mechanism such as the passive leakage from stem parenchyma cells adjacent to xylem vessels during necrosis or a more active mechanism such as the use of a type-III effector. However, VIRION simulations of scenario #4, the scenario closest to the experimental observations (Gerlin et al. 2021a), shows that, even with a transpiration limit, a moderate growth rate of *R. pseudosolanacearum* of 0.16h^−1^ can be reached up to 10^9^ CFU/g stem FW and a low growth rate above 0.01h^−1^ can be reached until 10^9.7^ CFU/g stem FW solely through biotrophic growth. Above 10^10^ CFU/g stem FW, a growth rate is still possible but very close to 0. 10^10^ CFU/g stem FW is thus likely the maximal bacterial load achievable in susceptible tomato stem. From these results we can conclude that xylem sap, when considered as the product of a continuous flow, is not poor and very high bacterial densities can be reached solely through biotrophic growth of the pathogen.

Even if xylem sap is richer than initially thought, resource hijacking mechanisms cannot be excluded. We thus also investigated how resource hijacking could contribute to *R. pseudosolanacearum* growth. We confirmed by simulation that this mechanism could theoretically be an effective pathogen strategy to boost its growth and compensate, for example, the effect of transpiration decline, since it allowed the pathogen to have a maximal growth rate up to 10^9.28^ CFU/g stem FW. We also confirmed that in the case of stem resource hijacking, this would primarily involve sugars and some amino acids, mainly glucose, sucrose, and proline. (Figure 3d). This could explain why Jacobs et al. (2012) and Hamilton et al. (2021) reported that mutants defective in sucrose were less competitive in plants. In addition, it could also explain that Gerlin et al. (2021a) has observed a putative increase in proline, valine, isoleucine, and threonine. Nevertheless, we also showed that this mechanism is probably dispensable for *R. pseudosolanacearum* since pathogen growth rate is intermediate from 10^8^ to 10^9^ (Gerlin et al. 2021a), contrary to model scenario #6's predictions. This also aligns with the observation that mutants defective in sugar assimilation still fully wilt tomato plants, with a slight delay (Jacobs et al. 2012; Gerlin et al. 2021a). We thus conclude that bacterial wilt can hijack plant resources, but the quantity of nutrients extracted remains significantly lower than the model's theoretical maximum.

Reducing the amount of glutamine—the major carbon source for the bacterium—in the xylem sap seems to be a better strategy for limiting the growth of *R. pseudosolanacearum* in the plant. However, such modifications would likely affect plant metabolism and phenotype even before impacting the pathogen, as our model tends to indicate. Nevertheless, it might be worthwhile to consider how plant species/plant cultivar or managing the plant's environment, such as nutrition or watering, could mitigate xylem sap flow and composition and thus minimize *R. pseudosolanacearum* growth in the plant.

While *R. pseudosolanacearum* appears to favor a fast consumption of resources, as its metabolic and growth capacities outcompete those of the plant, other plant pathogens might not exhibit this behavior but instead favor a reduced growth in xylem vessels. In particular, *Xylella fastidiosa*, although capable of metabolizing various xylem sap components (Gerlin et al. 2020), has very limited growth rates in vitro and *in planta*, and is described as adopting a self-limiting behavior (Roper et al. 2019; Gerlin et al. 2020). In these conditions, the plant would be able to dominate the trophic interaction and limit bacterial spread. This suggests that these pathogens would favor a long stay in xylem vessels at low bacterial densities. In this case, a coexistence with the plant takes place in which the bacterium is not competing for resources, and both the plant and the pathogen can remain metabolically active.

## Methods

### Construction of VIRION

The cell tomato (*solanum lycopersicum*) metabolic model from Gerlin et al., (2022) was used as a starting point. Reactions for iron, manganese, cobalt, molybdenum, and zinc assimilation, which were not represented in the initial metabolic model, were added as these metabolites were necessary for predicting *R. pseudosolanacearum* growth by using its GSMM (Peyraud et al. 2016). This addition, as well as other changes or improvements on putrescine metabolism and biomass, is fully described in Text S1.

From the single-cell tomato model, a multiorgan model was generated using the same procedure as in Gerlin et al. (2022). Briefly, using an in-house script, the metabolic network was replicated into 3 metabolic networks representing leaf, stem, and root with adequate biomass reaction for each organ. Exchange reactions were added to allow the exchange of metabolites between organs through xylem and phloem, considering weight ratios between the different organs. Xylem represented exchanges between root and aerial parts, while phloem represented exchanges between aerial parts and roots. Stem could exchange in both directions with the phloem. Contrary to our previous multiorgan metabolic model, stem could uptake elements from the xylem but could not provide elements to this compartment, except when mentioned. As in Gerlin et al. (2022), a transport cost was added for each exchange reactions except for water. Different physiological constraints (uptakes of water and minerals by roots only, no photosynthesis for root, limited photosynthesis for stem) were integrated to model the different roles of each organ as in Gerlin et al. (2022).

The in-house scripts also merged *R. pseudosolanacearum* metabolic network taken from Peyraud et al. (2016) to the multiorgan network, generating a 4-compartment model: leaf, stem, root, pathogen. The boundary metabolites were removed from the metabolic model of *R. pseudosolanacearum*, and external metabolites of the pathogen model were considered as xylem metabolites. To avoid dead-end metabolites, sink reactions were added allowing the exit of *R. pseudosolanacearum* metabolites excreted into the xylem and not present in healthy plant xylem sap. Simulations revealed very few sink reactions were used, and at very low fluxes. In addition, experimental data showed that few metabolites were enriched upon *R. pseudosolanacearum* infection in xylem sap (Lowe-Power et al. 2018a; Gerlin et al. 2021a). The weight ratio between *R. pseudosolanacearum* and the plant was implemented in the exchange reactions between the xylem and the bacterial periplasm and was modified to represent the different bacterial densities. Conversion of *R. pseudosolanacearum* weight into CFU per g of FW was estimated to follow 1 CFU/g stem FW = 1.10 · 10^−11^ g bacteria/1.52 g stem dry weight (DW), based on: (i) optical density (OD)/CFU and OD/g ratios from (Peyraud et al. 2016), (ii) FW/DW from Gerlin et al. (2022), (iii) the leaf/stem and stem/root ratios in tomato metabolic model (Gerlin et al. 2022), which impose relative contents of 1.52 g stem DW and 3.37 g leaf DW for 1 g root DW (Text S2).

### Calibration of *R. pseudosolanacearum* metabolic model for growth *in planta*

To determine growth, excretion, and assimilation capacities of *R. pseudosolanacearum* in the presence of xylem carbon sources, we used the data and model generated in Baroukh et al. (2025). In the study, a shake-flask growth experiment was performed in a minimal medium supplemented with a mixture of organic substrates whose relative concentrations mimics a tomato xylem. Growth, substrate assimilation, and product excretion were monitored and used to determine uptake, excretion, and growth rates among this combination of carbon sources. Glutamine and glucose assimilation followed Monod-type kinetics and were driving growth, while other substrate assimilation rates were proportional to the assimilation of glutamine and glucose. From the Monod kinetics, 2 maximal assimilation rates, one for glutamine and one for glucose, were implemented as a constraint bound in the model:


$$Gln_{ass_{rate}}\leq k_{gln}*\mu_{max_{gln}}*\frac{\left[Gln\right]}{[Gln]+K_{s_{gln}}}*1000*24,$$

$$Gluc_{ass_{rate}}\leq k_{gluc}*\mu_{max_{gluc}}*\frac{\left[Gluc\right]}{[Gluc]+K_{s_{gluc}}}*1000*24,$$

with:

-
$Gln_{ass_{rate}}$ the assimilation rate of glutamine in the model (mmol·gB^−1^·d^−1^), $k_{gln}$ (mmol·mgB^−1^) the quantity of glutamine necessary to yield 1 mg of biomass, $\mu_{\begin{array}{c} \mathrm{ma}x_{gln} \end{array}}$ (h^−1^) the maximal growth rate on glutamine, $K_{Sgln}$ (mM) the semisaturation growth term on glutamine, [*Gln*] (mM) the concentration of glutamine in tomato xylem sap as measured Gerlin et al. (2021a);
-
$Gluc_{ass_{rate}}$ the assimilation rate of glucose in the model (mmol·gB^−1^·d^−1^), $k_{gluc}$ (mmol·mgB^−1^) the quantity of glucose necessary to yield 1 g of biomass, $\mu_{\begin{array}{c} \mathrm{ma}x_{gluc} \end{array}}$ (h^−1^) the maximal growth rate on glucose, $K_{Sgluc}$ (mM) the semisaturation growth term on glucose, [*Gluc*] (mM) the concentration of glucose in tomato xylem sap as measured in Gerlin et al. (2021a).

From all the other substrates, to ensure assimilation only when glucose or glutamine were assimilated, a constraint proportional to glucose and glutamine rate was added:


$$os_{ass_{rate}}\leq \frac{k_{OS}}{k_{gln}}*Gln_{ass_{rate}}+\frac{k_{OS}}{k_{gluc}}*Gluc_{ass_{rate}},$$

with $os_{ass_{rate}}$ (mmol·gB^−1^·d^−1^) the assimilation rate of the other substrate (phenylalanine, valine, leucine, arginine, sucrose, tyrosine, proline, lysine, threonine, isoleucine, asparagine). All values were taken from Baroukh et al. (2025).

The growth-associated and non-growth-associated ATP maintenance terms were calibrated so that *R. pseudosolanacearum* growth rate prediction was in agreement with experimental data measured in Baroukh et al. (2025).

### Sequential FBAs

Modeling of the pathosystem was performed using 4 successive FBAs with different objective functions.

1. Minimization of photon uptake

This first FBA was performed on the plant alone. It minimized photon uptake while leaf, stem, and root growth rates were constrained to their experimental values taken from Gerlin et al. (2022). Minimizing photon uptake allowed for estimating the physiological capacities (photosynthesis, iron, nitrogen uptake) of a healthy plant.

2. Maximization of the pathogen growth

The second FBA was performed on the plant–pathogen model. It maximized the pathogen biomass growth while the photon uptake rate was constrained to the value found in FBA 1 (representing healthy plant photosynthetic capacities). This constraint avoided having an infected plant with a higher photosynthesis rate than a healthy plant. The leaf and stem growth rates were relaxed, to model the fact that *R. pseudosolanacearum* resource acquisition and proliferation can hinder the growth of the aerial part, as observed experimentally (Gerlin et al. 2021a). Root growth rate remained constrained as it was found experimentally that root growth was not impacted by *R. pseudosolanacearum* presence (Gerlin et al. 2021a).

In this FBA, nitrogen or iron uptake rate could also be constrained to the ones of a healthy plant as found in FBA 1. Transpiration rate of the plant could also be constrained. Indeed, measurements of transpiration during tomato–*R. pseudosolanacearum* interaction revealed a decrease of transpiration when the pathogen is present at a density higher than 10^7^ CFU/g of stem FW (Gerlin et al. 2021a). We estimated, thanks to a linear regression, the following linear rule for transpiration decrease:


$$\mathrm{reduction}\_\mathrm{flow}\_\mathrm{coeff}=-0.2746*[log_{10}CFU/gstemFW]+2.8764.$$

As xylem fluxes of matter depend on the transpiration rate, the upper bound of each flux exchanged between the root and the xylem (v_Exch_r_xyl_i_) was constrained by the value found in FBA1 (vFBA1_Exch_r_xyl_i_), reduced by the reduction_flow_coeff, when the bacterial density exceeded 10^7^ CFU/g stem FW:


$$\mathrm{If}[\log_{10}\;CFU/gstemFW]>7\mathrm{then}\;v_{\mathrm{Exch}\_r\_\mathrm{xyl}\_i\;\text{£}}\mathrm{vFBA}1_{\mathrm{Exch}\_r\_\mathrm{xyl}\_i*}\mathrm{reduction}\_\mathrm{flow}\_\mathrm{coeff}.$$

3. Maximization of the growth of the aerial parts

The third FBA was performed on the plant–pathogen model. It maximized biomass growth of the stem and the leaves of the plant, while the pathogen growth rate was constrained to the value found in FBA 2. A constant ratio between leaf and stem biomass was imposed to ensure balanced growth of both the stem and the leaves. This FBA determined the growth of the aerial parts achievable in the presence of *R. pseudosolanacearum*, since the pathogen used part of xylem nutrients.

4. Minimization of the sum of absolute fluxes

The fourth and final FBA was performed on the plant–pathogen model. It minimized the sum of all fluxes of the model to ensure a parsimonious enzyme usage in both organisms as well as avoid the presence of stoichiometric balanced cycles. The leaf and stem growth rates were constrained to the values found in FBA 3.

### Software requirements

Simulations were performed with Python 3 scripts, using the open access libraries lxml and pandas, and the linear programming solver CPLEX Python API, developed by IBM. The scripts used to build the GSMMs and perform all the simulations can be found on GitHub at this link: https://github.com/cbaroukh/VIRION.

An academic or commercial license of CPLEX Python API (ILOG CPLEX Optimization Studio) is mandatory to run the scripts. The academic license is free for academic institutions. The basic “free Community Edition” version of CPLEX is not suitable and will provide an error message.

## Supplementary Material

kiag428_Supplementary_Data

## Data Availability

All data supporting the findings of this study are available within the paper and within its Supplementary materials. The scripts used to perform all the simulations, as well as the GSMMs, can be found on GitHub at this link: https://github.com/cbaroukh/VIRION.
