# Supplementary material for: A genome-scale metabolic model of a pathosystem sheds light on bacterial wilt
Source: Plant Physiol. 2026 Jul 1;201(4):kiag428. doi: 10.1093/plphys/kiag428 (PMC13464506; doi:10.1093/plphys/kiag428)
Supplement: Supplementary file 1 [file kiag428_supplementary_data.pdf]

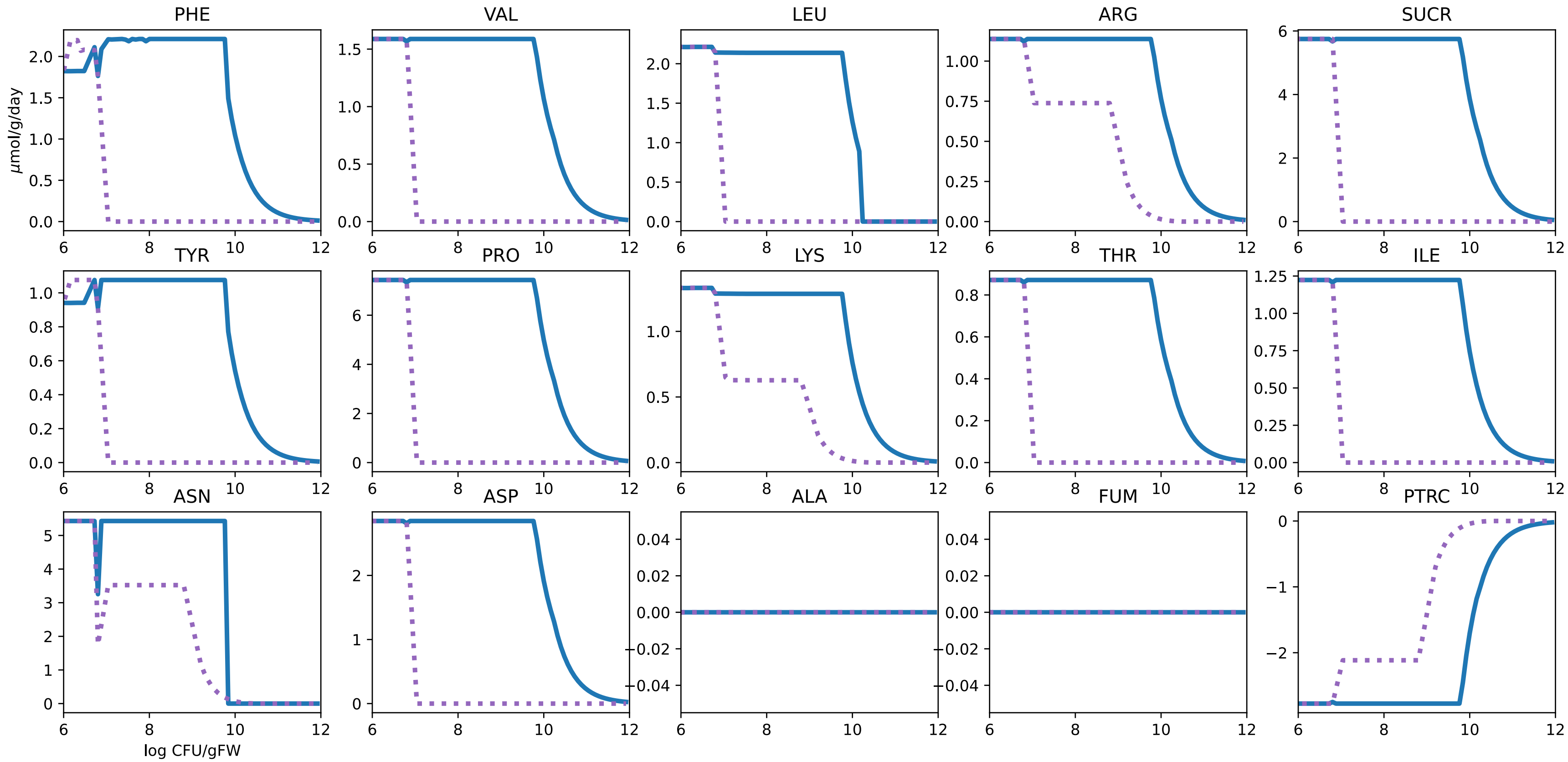

Supplementary Figure S1. *R. pseudosolanacearum* uptake rates for different xylem metabolites depending on the bacterial density and the limiting factors. g: g of *R. pseudosolanacearum* dry weight. CFU: colony forming units. PHE: phenylalanie, VAL: valine, LEU: leucine, ARG: arginine, SUCR: sucrose, TYR: tyrosine, PRO: proline, LYS: lysine, THR: threonine, ASN: asparagine, ASP: aspartate, ALA: alanine, FUM: fumarate, PTRC: putrescine. Plain blue line: scenario #1, #2 and #3 (no transpiration constraint in the model). Dotted purple line: scenario #4 and #5 (transpiration constraint in the model). g here means g of dry weight of *R. pseudosolanancearum.*

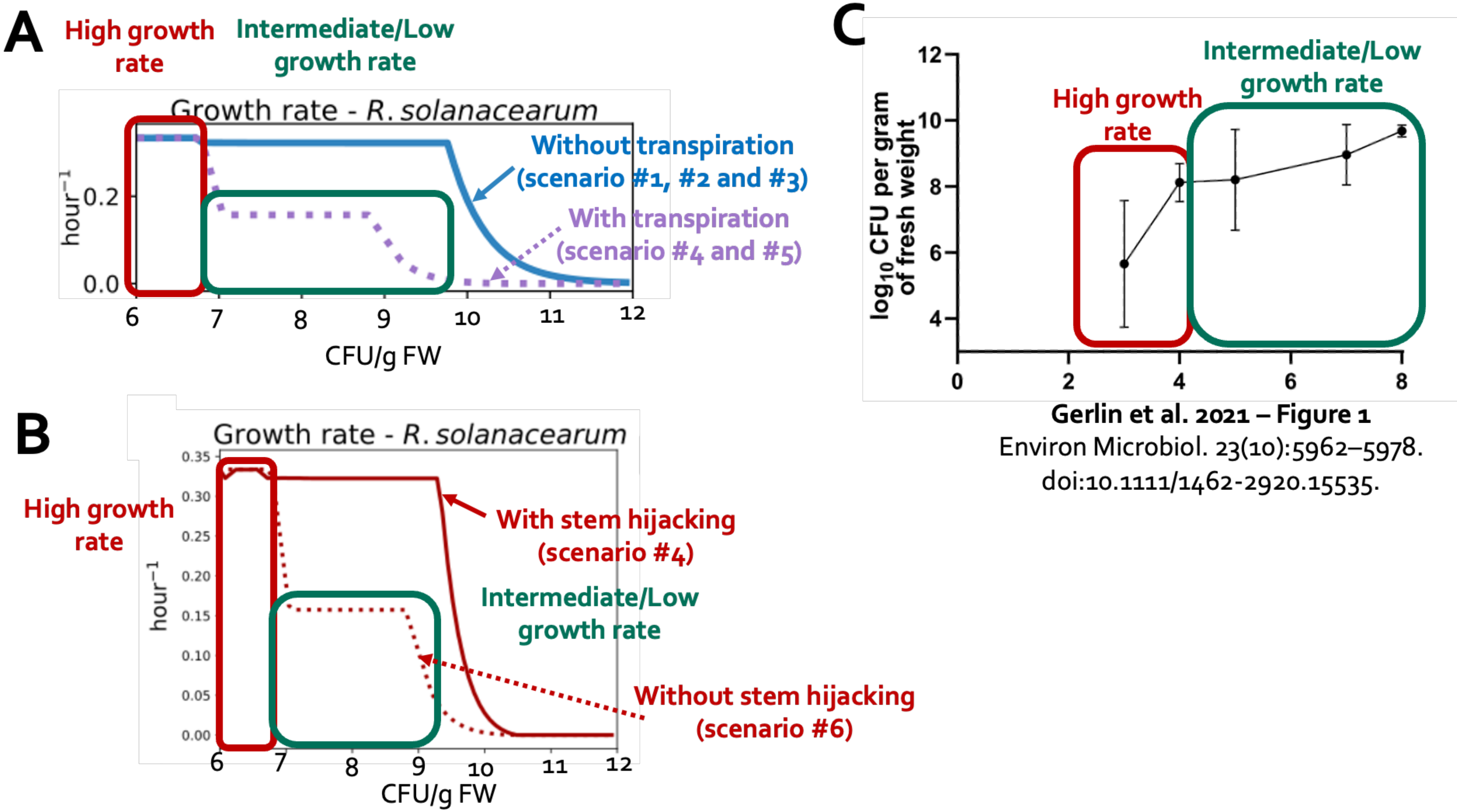

Supplementary Figure S2. Comparison of models' predictions for different scenarios (#1 to #6) to experimental data of Gerlin et al. (2021). A. *R. pseudosolanacearum* growth prediction for bacterial densities ranging from 10^6 to 10^{12} CFU/g stem fresh weight for scenarios #1 to #5. B. *R. pseudosolanacearum* growth prediction for bacterial densities ranging from 10^6 to 10^{12} CFU/g stem fresh weight for scenarios #4 and #6. C. Growth of *R. pseudosolanacearum* experimentally measured in tomato stem in Gerlin

et al. (2021). Experimental measurements highlight a high growth rate up to 10^8 CFU/g stem fresh weight, an intermediate/low growth rate from 10^8 CFU/g stem fresh weight to 10^{10} CFU/g stem fresh weight and a maximal bacterial density just below 10^{10} CFU/g stem fresh weight. FW: fresh weight.

Supplementary Table S1

Experimental constraints used to calibrate the multi-organism metabolic model.

Variable	Constraint	Value	Note	References
leaf growth rate (day^{-1})	Lower bound	0.21		https://doi.org/10.1093/plphys/kiab548
stem growth rate (day^{-1})	Lower bound	0.26		https://doi.org/10.1093/plphys/kiab548
root growth rate (day^{-1})	Lower bound	0.17		https://doi.org/10.1093/plphys/kiab548
organic uptake, pathogen (mmol/g/day)	Upper bound	value in the macroscopic model of Ralstonia		https://doi.org/10.1111/nph.20216
pathogen:plant ratio (cells per g of plant fresh weight)	Stoichiometric coefficient between plant and xylem, and xylem and pathogen	10^6 - 10^{12}	Range of experimentally plausible values, based on experimental measurements	https://doi.org/10.1111/1462-2920.15535
transpiration reduction	Upper bound of 'root to xylem' exchange fluxes	*(-0.2746* [log$_{10}$ CFU.g^{-1} FW] + 2.8764)* of		https://doi.org/10.1111/1462-2920.15535

		uninfected root to xylem flux from FBA 1		
leaf:stem ratio (g/g)	Stoichiometric coefficient between leaf and stem biomass fluxes	2.2	To allow both leaf and stem biomass maximization in FBA 3	https://doi.org/10.1093/plphys/kiab548

Supplementary text S1

Modifications of tomato metabolic model to build a whole plant – pathogen model

1. Determination of iron concentration in tomato plant from literature for integration in biomass

Iron was initially absent from tomato biomass. Regarding its importance in plant – *Ralstonia* interaction (Bhatt and Denny, 2004), we added it to organ biomass reactions (R_BIOMASS_ROOT, R_BIOMASS_STEM, R_BIOMASS_LEAF). We determined the coefficient value in the equation following the procedure below.

- **From Twyman, 1959 (Twyman, 1959)**

Mean concentration of iron in dry leaf tissue of tomato plants:
[Fe] = 144 ppm at the highest iron supply (Table 2)
[Fe] = 144 ppm = 144 mg Fe/kg leaf DW

Note: at the lowest iron supply, the value remains close (112 mg Fe/kg leaf DW).

- **From Carpena et al., 1988 (Carpena et al., 1988)**

Cultivar	Vermone	Marglobe
Nutrient consumption (mg Fe/plant) – Table 3	301	298
Yield, plant weight (g/plant) – Table 4	3054	2613
Yield, fruit weight (g/plant) – Table 4	6648	3930
Yield, plant dry weight (g DW/plant)*	432	370
Yield, fruit dry weight (g DW/plant)**	398	235
Total dry weight (g DW/plant)	830	605
Total dry weight (kg DW/plant)	0.830	0.733
**[Fe] (mg Fe/kg DW)*** **	**363**	**406.5**

*Using FW/DW ratio of 7.07 (Gerlin et al., 2022)

**Using FW/DW ratio of 16.7 (6% of dry content) (Cozzolino et al., 2023)

***[Fe] (mg Fe/kg DW) = Nutrient consumption (mg Fe/plant)/Total dry weight (kg DW/plant)

➔ **Integration of iron concentration in tomato model**

The range estimated from literature is **144 – 406.5 mg Fe/kg DW** in a tomato plant.
Biomass equation represents 1 g of organ DW, corresponding to 1.44 – 4.07 10^{-4} g Fe.
Using the atomic weight of Fe (55.845 g/mol), it represents 2.58 – 7.28 10^{-6} mol Fe.
Biomass equation is expressed in µmol/gDW, so the range of values to integrate in the model is: **2.58 – 7.28 µmol/gDW.** Iron is majorly found in and between plant organs as Fe2+ (M_fe2_c) (Briat et al., 2007), so iron will has been incorporated in biomass equation as M_fe2_c.
Here, we did not take into account the organ specificity of iron concentration and add the same value in leaf, stem and root. We integrated the highest value to mimic a maximal requirement. Simulations presented in the paper show that even this high value has poor impact on the interaction.

2. Addition of minerals required for pathogen growth

Molybdate (M_mobd_c), manganese (M_mn_c) and cobalt (M_cobalt_c) are present in *R. pseudosolanacearum* biomass equation while they were not modeled in plant initial metabolic model (Sl2183).
We added:

- **For molybdate**

The internal metabolite M_mobd_c was already present. We added the boundary metabolite M_mobd_b and the uptake reaction R_EX_mobd_c, written as follow:
M_mobd_b <-> M_mobd_c

- **For manganese**

We added the internal and boundary metabolites M_mn2_b and M_mn2_c and the uptake reaction R_EX_mn2_c, written as follow:
M_mn2_b <-> M_mn2_c

- **For cobalt**

We added the internal and boundary metabolites M_cobalt2_b and M_cobalt2_c and the uptake reaction R_EX_cobalt2_c, written as follow:
M_ cobalt2_b <-> M_ cobalt2_c

3. Corrections of amino acid coefficients in biomass equation

The coefficients for some amino acids were inverted in the previous files of tomato model published in 2022 (Gerlin et al., 2022). While it does not have significant impact on the simulations, either here or in the previous work, we rewrote the coefficients for the following amino acids: M_cys__L_c, M_ala__B_c, M_his__L_c, M_ile__L_c, M_leu__L_c, M_lys__L_c with the correct values from the list of biomass components (Supp File 1 of Gerlin et al. 2022 (Gerlin et al., 2022), sheets 3, 4, 5).

4. Putrescine metabolism

We manually examined putrescine catabolism in Sl2183, as our initial model was not representing putrescine catabolism. We looked at canonical putrescine catabolism in plants in literature (Rastogi and Davies, 1990) and databases (https://pmn.plantcyc.org/pathway?orgid=ARA&id=PWY-2).
We found that the first reaction of putrescine catabolism, putrescine oxidase* (EC 1.4.3.10) was already included in our model (converting putrescine into 4-aminobutanoate), but we missed the following reaction, aminobutyraldehyde dehydrogenase** (EC 1.2.1.19), converting 4-aminobutanoate into GABA. We found two orthologs (SOLYC06G071290 OR SOLYC03G113800) to the A. thaliana gene (ALDH10A9 / AT3G48170) annotated as aminobutyraldehyde dehydrogenase in BioCyc. This provides evidence for the completeness of the pathway in tomato, and we thus added aminobutyraldehyde dehydrogenase in the modeL

*Putrescine oxidase
R_PTRCOX1 http://bigg.ucsd.edu/universal/reactions/PTRCOX1 in BiGG
PUTRESCINE-OXIDASE-RXN https://biocyc.org/reaction?orgid=META&id=PUTRESCINE-OXIDASE-RXN in BioCyc
Reaction in BiGG: M_h2o_c + M_o2_c + M_ptrc_c -> M_4abutn_c + M_h2o2_c + M_nh4_c

**Aminobutyraldehyde dehydrogenase
R_ABUTD http://bigg.ucsd.edu/models/iJO1366/reactions/ABUTD in BiGG
AMINOBUTDEHYDROG-RXN https://biocyc.org/reaction?orgid=META&id=AMINOBUTDEHYDROG-RXN in BioCyc
Reaction in BiGG: M_4abutn_c + M_h2o_c + M_nad_c -> M_4abut_c + 2.0 M_h_c + M_nadh_c

References

Bhatt, G., Denny, T.P., 2004. *Ralstonia solanacearum* Iron Scavenging by the Siderophore Staphyloferrin B Is Controlled by PhcA, the Global Virulence Regulator. J. Bacteriol. 186, 7896–7904. https://doi.org/10.1128/JB.186.23.7896-7904.2004

Briat, J.-F., Curie, C., Gaymard, F., 2007. Iron utilization and metabolism in plants. Curr. Opin. Plant Biol. 10, 276–282. https://doi.org/10.1016/j.pbi.2007.04.003

Carpena, O., Masaguer, A., Sarro, M.J., 1988. Nutrient uptake by two cultivars of tomato plants. Plant Soil 105, 294–296. https://doi.org/10.1007/BF02376796

Cozzolino, E., Salluzzo, A., Piano, L.D., Tallarita, A.V., Cenvinzo, V., Cuciniello, A., Cerbone, A., Lombardi, P., Caruso, G., 2023. Effects of the Application of a Plant-Based Compost on Yield and Quality of Industrial Tomato (Solanum lycopersicum L.) Grown in Different Soils. Appl. Sci. 13, 8401. https://doi.org/10.3390/app13148401

Gerlin, L., Cottret, L., Escourrou, A., Genin, S., Baroukh, C., 2022. A multi-organ metabolic model of tomato predicts plant responses to nutritional and genetic perturbations. Plant Physiol. 188, 1709–1723. https://doi.org/10.1093/plphys/kiab548

Rastogi, R., Davies, P.J., 1990. Polyamine Metabolism in Ripening Tomato Fruit 1: I. Identification of Metabolites of Putrescine and Spermidine. Plant Physiol. 94, 1449–1455. https://doi.org/10.1104/pp.94.3.1449

Twyman, E.S., 1959. The effect of iron supply on the yield and composition of leaves of tomato plants. Plant Soil 10, 375–388. https://doi.org/10.1007/BF01666211

Supplementary Text S2

Integration of bacterial density in VIRION

Numerical values used

$1.0157 \cdot 10^{12}$ CFU *Rs*/L = 1 OD	Value from (Peyraud et al., 2016)<br>Linear regression from S4 Table.
0.414 g *Rs*/L = 1 OD	Value from (Peyraud et al., 2016)<br>Materials and Methods.
g fresh weight / g dry weight [*Sl* stem] = 17.7	Value from (Gerlin et al., 2022)<br>Figure 2.

g / CFU [*Rs*] = 0.414 g *Rs*/L / $1.0157 \cdot 10^{12}$ CFU *Rs*/L = $4.076 \cdot 10^{-13}$

1 cfu/g stem FW = 1·(g fresh weight / g dry weight [*Sl* stem]) = 17.7 **cfu/g stem DW**

In VYTOP model, the relative proportions are: 1.52 g stem DW and 3.37 g leaf DW for 1 g root DW. The bacterial density must be determined for 1.52 g stem DW.

1 cfu/g stem FW = 1.52 · 17.7 **cfu/1.52 g stem DW** = 26.904 **cfu/1.52 g stem DW**
1 cfu/g stem FW = 26.904 · (g / CFU [*Rs*]) **g bact / 1.52 g stem DW**
1 cfu/g stem FW = 26.904 · $4.076.10^{-13}$ **g bact / 1.52 g stem DW**
1 cfu/g stem FW = $1.10 \cdot 10^{-11}$ **g bact / 1.52 g stem DW**

Rs: *Ralstonia pseudosolanacearum* GMI1000
Sl: *Solanum lycopersicum* M80

References

Gerlin, L., Cottret, L., Escourrou, A., Genin, S., Baroukh, C., 2022. A multi-organ metabolic model of tomato predicts plant responses to nutritional and genetic perturbations. Plant Physiol. 188, 1709–1723. https://doi.org/10.1093/plphys/kiab548

Peyraud, R., Cottret, L., Marmiesse, L., Gouzy, J., Genin, S., 2016. A Resource Allocation Trade-Off between Virulence and Proliferation Drives Metabolic Versatility in the Plant Pathogen Ralstonia solanacearum. PLoS Pathog. 12, e1005939. https://doi.org/10.1371/journal.ppat.1005939
